# α4-Containing GABA_A_ Receptors on DRD2 Neurons of the Nucleus Accumbens Mediate Instrumental Responding for Conditioned Reinforcers and Its Potentiation by Cocaine

**DOI:** 10.1523/ENEURO.0236-23.2023

**Published:** 2023-08-28

**Authors:** Tom Macpherson, Claire I. Dixon, Jonathan Robertson, Marsha M. Sindarto, Patricia H. Janak, Delia Belelli, Jeremy J. Lambert, David N. Stephens, Sarah L. King

**Affiliations:** 1 Sussex Neuroscience, School of Psychology, University of Sussex, Brighton BN1 9QG, United Kingdom; 2 Laboratory for Advanced Brain Functions, Institute for Protein Research, Osaka University, Suita 565-0871, Japan; 3 Department of Psychological and Brain Sciences, Krieger School of Arts and Sciences, Johns Hopkins University, Baltimore, Maryland 21218; 4 Division of Neuroscience, Medical Research Institute, Ninewells Hospital & Medical School, University of Dundee, Dundee DD1 9SY, United Kingdom

**Keywords:** GABA_A_ receptors, nucleus accumbens, reward, cocaine, THIP

## Abstract

Extrasynaptic GABA_A_ receptors (GABA_A_Rs) composed of α4, β, and δ subunits mediate GABAergic tonic inhibition and are potential molecular targets in the modulation of behavioral responses to natural and drug rewards. These GABA_A_Rs are highly expressed within the nucleus accumbens (NAc), where they influence the excitability of the medium spiny neurons. Here, we explore their role in modulating behavioral responses to food-conditioned cues and the behavior-potentiating effects of cocaine. α4-Subunit constitutive knock-out mice (α4^−/−^) showed higher rates of instrumental responding for reward-paired stimuli in a test of conditioned reinforcement (CRf). A similar effect was seen following viral knockdown of GABA_A_R α4 subunits within the NAc. Local infusion of the α4βδ-GABA_A_R-preferring agonist THIP (4,5,6,7-tetrahydroisoxazolo[5,4-c]pyridin-3-ol; Gaboxadol) into the NAc had no effect on responding when given alone but reduced cocaine potentiation of responding for conditioned reinforcers in wild-type, but not α4^−/−^ mice. Finally, specific deletion of α4-subunits from dopamine D2, but not D1, receptor-expressing neurons (DRD2 and DRD1 neurons), mimicked the phenotype of the constitutive knockout, potentiating CRf responding, and blocking intra-accumbal THIP attenuation of cocaine-potentiated CRf responding. These data demonstrate that α4-GABA_A_R-mediated inhibition of DRD2 neurons reduces instrumental responding for a conditioned reinforcer and its potentiation by cocaine and emphasize the importance of GABAergic signaling within the NAc in mediating the effects of cocaine.

## Significance Statement

This article combines genetic and pharmacological interventions to uncover a critical role for α4-containing GABA_A_ receptors in the nucleus accumbens in instrumental responding for conditioned reinforcers and its potentiation by cocaine, a behavioral phenomenon thought to contribute to reward-seeking behavior. These findings represent an important advancement in our understanding of the neural mechanisms underlying the reinforcing effects of conditioned stimuli and the role of the GABAergic system in this process.

## Introduction

GABA_A_ receptors (GABA_A_Rs) are a family of heteropentameric cys-loop ligand-gated chloride channels that function as the major mediators of inhibition in the mammalian CNS. More than 95% of the neurons in the striatum are GABAergic, and as such, GABA_A_Rs are likely to play an important role in modulating signaling in basal ganglia reward pathways, including GABAergic medium spiny neurons (MSNs) of the nucleus accumbens (NAc) (Robison and Nestler, 2011; Macpherson et al., 2014; Stephens et al., 2017). The NAc is critically involved in pavlovian associative processes, including conditioned reinforcement (CRf), whereby an environmental conditioned stimulus (CS), which previously held no intrinsic value, acts to reinforce instrumental behavior following its association with a biologically significant unconditioned stimulus (US) and thereby acquires motivational value (Mackintosh, 1974; Kelley and Delfs, 1991; Everitt et al., 2001). This phenomenon is thought to contribute to natural and drug reward-seeking behaviors and is known to be potentiated by psychostimulant drugs, including cocaine (Robbins, 1975; Cador et al., 1991; Parkinson et al., 1999; Everitt and Robbins, 2005). Although the importance of NAc dopamine availability for CRf and its potentiation by psychostimulants has been well established (Hill, 1970; Robbins, 1975, 1978; Beninger et al., 1980; Taylor and Robbins, 1986), considerably less is known about the role of GABA signaling in these processes.

Within the NAc, synaptic α2-GABA_A_Rs mediate phasic inhibition and influence locomotor sensitization to cocaine, as well as cocaine-potentiation of responding for CRf (Morris et al., 2008; Dixon et al., 2010). Additionally, extrasynaptic α4βδ-GABA_A_Rs mediate a tonic inhibitory conductance in the NAc, which influences the excitability of MSNs (Maguire et al., 2014). Our previous finding (Maguire et al., 2014) that activation of α4βδ GABA_A_Rs using the selective δ-GABA_A_R agonist THIP (4,5,6,7-tetrahydroisoxazolo[5,4-c]pyridin-3-ol; Gaboxadol) attenuates cocaine potentiation of cocaine-conditioned place preference (cocaine-CPP) indicates that these receptors may play a broader role in motivational processes.

MSNs within the striatum are typically divided into two subpopulations based on their expression of dopamine receptor subtypes, releasable peptides, and their axonal projection targets. DRD1-MSNs express dopamine D1 receptors (D1Rs), dynorphin, and substance P, whereas DRD2-MSNs express D2 receptors (D2Rs) and enkephalin (Gerfen et al., 1990, 1991; Gerfen, 1992). These two subpopulations are suggested to act in an opposing manner to control limbic functions, with DRD1-MSNs and DRD2-MSNs mediating reward-related and aversion/error-related learning, respectively (Macpherson et al., 2014, 2021; Hikida et al., 2016; Nakanishi et al., 2014). Indeed, activity in NAc DRD1-MSNs is reported to mediate reinforcement, pavlovian conditioned approach, instrumental responding for natural and drug rewards, and cocaine sensitization (Hikida et al., 2010; Lobo et al., 2010; Gore and Zweifel, 2013; Creed et al., 2016; Macpherson and Hikida, 2018; Attachaipanich et al., 2023), whereas activity in NAc DRD2-MSNs has been reported to be necessary for behavioral flexibility and the suppression of cocaine-induced place preference and motivation to seek cocaine (Macpherson et al., 2016, 2022; Lobo et al., 2010; Bock et al., 2013). Interestingly, this proposed opposing role of NAc DRD1-MSNs and DRD2-MSNs has been challenged by evidence that both MSN types are able to control reward and aversion (Soares-Cunha et al., 2019) and that a subtype of NAc DRD1-MSNs expressing tachykinin 2 are able to negatively regulate cocaine place preference (Zhao et al., 2022), indicating that the roles of NAc MSNs in limbic control may be more nuanced than originally thought (Soares-Cunha et al., 2016b). 

Here, using a combination of genetic deletion of α4-GABA_A_Rs and α4-subunit viral knockdown in mice, alongside pharmacological activation of α4βδ GABA_A_Rs by the agonist THIP, we demonstrate that α4βδ-GABA_A_R-mediated tonic inhibition in the NAc suppresses behavioral responses to natural reward-paired stimuli, as well as their potentiation by cocaine. Then, using cell-specific GABA_A_R α4-subunit knock-out mice alongside intra-NAc infusion of THIP, we reveal that these effects occur via α4βδ-GABA_A_R inhibition of DRD2, but not DRD1, neurons. Our data suggest that α4βδ-GABA_A_Rs on NAc DRD1 and DRD2 neurons mediate different aspects of reward-related behaviors.

## Materials and Methods

### Production of mice

Constitutive α4-subunit knock-out mice were produced at Sussex University by crossing floxed α4 mice (B6.129-*Gabra4^tm1.2Geh^*/J (The Jackson Laboratory), with loxP sites flanking the first coding exon (exon3) of the gabra4 gene, with Cre-recombinase-expressing transgenic mice (Chandra et al., 2006; Maguire et al., 2014). Heterozygote mice at Sussex were maintained on a C57BL/6J background, and experimental wild-type (WT) mice and constitutive α4-subunit knock-out mice (α4^−/−^) generated from subsequent heterozygote matings. Conditional α4 knock-out mice with the deletion localized to DRD1 or DRD2 neurons, respectively (α4^D1−/−^ or α4^D2−/−^), were created by crossing the floxed α4 mice with BAC transgenic mice expressing Cre-recombinase under the control of either the dopamine receptor DRD1A or DRD2 gene [B6.FVB(Cg)-Tg(Drd1a-cre)EY266Gsat/Mmucd and B6.FVB(Cg)-Tg(Drd2-cre)ER44Gsat/Mmucd, respectively] as described in Maguire et al. (2014). Experimental mice were created by mating homozygote floxed a4 mice, with one of the pair also carrying the CRE transgene generating cell-specific knock-out or floxed WT controls. All mice were on a C57BL/6J background. C57BL/6J male mice (6–8 weeks) were purchased from Charles River Laboratories for the viral knock-down studies.

### Animals

Male and female (counterbalanced across conditions, data were grouped as no significant main effect or interactions of sex were observed) experimental mice, weighing between 20 and 30 g, were housed in groups of two to three. Body weights were maintained at ∼85% of free-feeding weight by the provision of a limited amount of standard lab chow (B&K Feeds) ∼2 h after completion of daily experiments. Water was available *ad libitum.* A 12 h light/dark cycle was used (lights on at 7:00 A.M.) with the holding room temperature maintained at 21 ± 2°C and humidity at 50 ± 5%. All injections, infusions, and behavioral testing were performed between 2:00 P.M. and 5:00 P.M. All procedures were conducted in accordance with the United Kingdom Animals (Scientific Procedures) Act 1986, following ethical review by the University of Sussex Ethical Review Committee.

### Stereotaxic cannulation

Mice anesthetized with isoflurane were implanted stereotaxically with bilateral guide cannulae (26 gauge) aimed at NAc (coordinates, AP 1.34, L ± 1.00, DV −3.20). Following surgery mice were singly housed and allowed to recover for 7 d. A steel infuser (33 gauge) connected via polyvinyl tubing to a (5 μl) Hamilton Gastight Syringe was used to infuse 1 μl (0.5 μl per side) of either saline or THIP bilaterally 1 mm beyond the guide cannulae, across 90 s, then left to settle for 90 s before infusers were removed. Following the completion of experiments, 0.2 ul of black ink was infused into each cannula. Mice were anaesthetized with pentobarbitol sodium and phenytoin sodium solution (Euthasol), then transcardially perfused with 4% paraformaldehyde (Sigma-Aldrich) and brains removed. Following submersion in 30% sucrose in PBS for 72 h, brains were sliced at 50 μm on a microtome and infusion locations were verified by eye with brightfield microscopy and recorded on a brain atlas of the NAc (Figs. 3*A*, 4*A*,*E*). Data from mice in which infusion sites were found to be outside the NAc were removed from analysis. The area of THIP infusion spread was estimated to be an ∼1.0–1.5 mm diameter sphere based on histologic analysis after intracranial infusion of a saturated solution of Fast Green dye at the same volume and infusion rate.

### Stereotaxic viral infusion

Mice anesthetized with isoflurane were stereotaxically infused with adenoviruses carrying an shRNA designed to knock down α4 (Ad-shα4) or a scrambled sequence (Ad-NSS) as well as expressing GFP under control of the CMV promotor, as previously described and validated in Rewal et al. (2009), bilaterally into the NAc (coordinates AP 1.34, L ± 1.40, DV −4.20). A steel infuser (33 gauge) connected via polyvinyl tubing to a (5 μl) Hamilton Gastight Syringe was used to infuse 1 ul (0.5 μl per side) of virus at a rate of 0.2 μl/min for 5 min then left to settle for an additional 5 min. Following surgery mice were singly housed and allowed to recover for 7 d. Following the completion of experiments, the location of the viral infusion was confirmed using immunofluorescence for GFP.

### CRf responding

**Figure 1.**
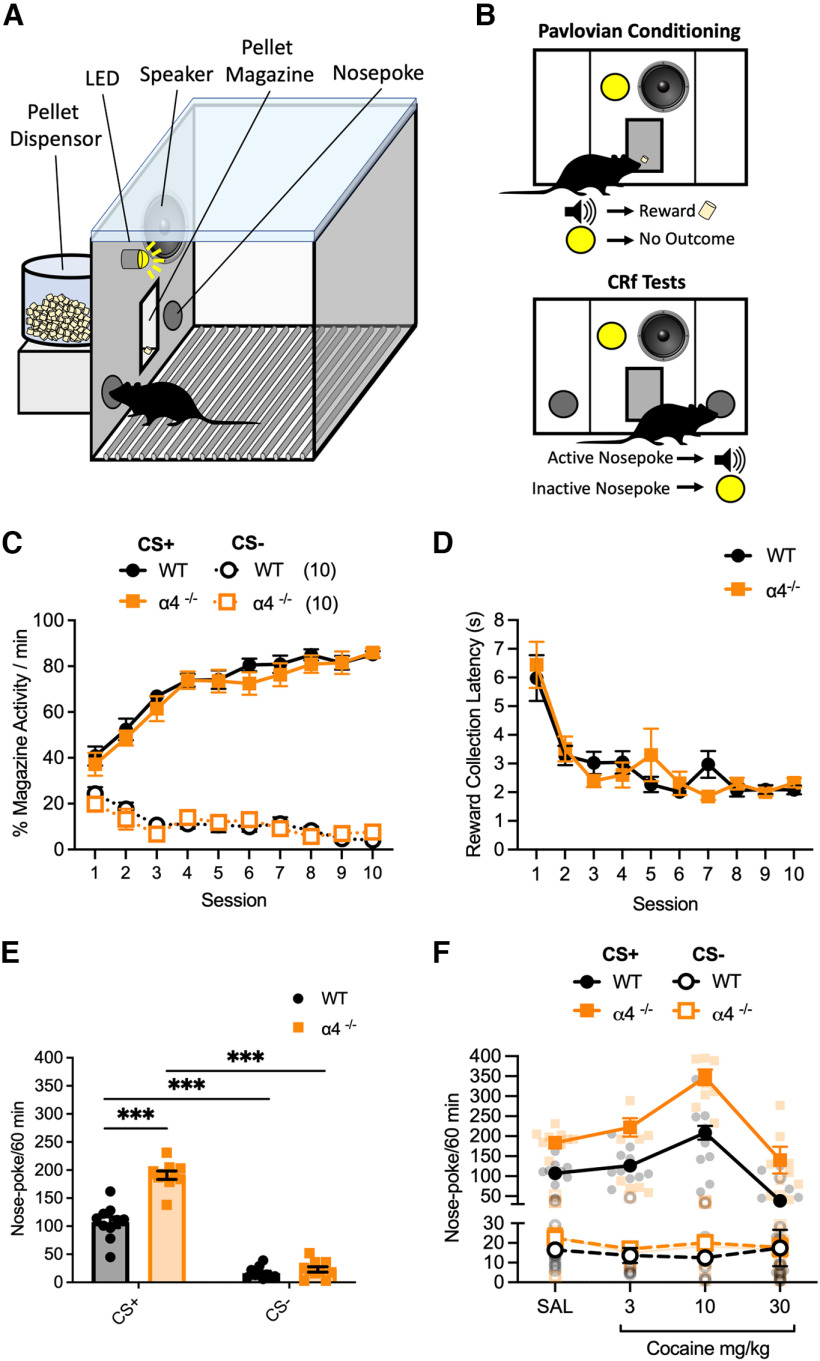
Instrumental responding for a conditioned reinforcer and its potentiation by cocaine in WT and α4^−/−^ mice. ***A***, Schematic of the operant chamber apparatus used for pavlovian conditioning and conditioned reinforcement tests. ***B***, Schematic of the procedures for pavlovian conditioning and conditioned reinforcement tests. ***C***, In pavlovian training, WT (*n* = 10) and α4^−/−^ (*n* = 10) mice learned the association between the cue (CS+) and delivery of a food reward at the same rate as indicated by increasing and decreasing numbers of magazine entries during the CS+ and CS−, respectively, over the course of 10 training sessions. ***D***, The latency in seconds to collect the pellet reward during pavlovian training decreased across the 10 training sessions, with no difference observed between WT and α4^−/−^ mice. ***E***, In a conditioned reinforcement responding test, both genotypes preferentially responded on a nose poke that led to CS+ presentations, compared with a CS− paired nose poke. However, α4^−/−^ mice made significantly more CS+ paired nose-poke responses than W mice. ***F***, At a 10 mg/kg dose, cocaine was able to potentiate CRf responding for the CS+ to a similar degree in WT and α4^−/−^ mice. Data are presented as mean ± SEM; ***p < 0.001, *post hoc* Bonferroni multiple comparisons.

Following food deprivation to maintain ∼85% of baseline body weight, animals were trained in mouse operant chambers (Med Associates), each housed within a light-resistant, sound-attenuating cubicle (Fig. 1*A*). Mice underwent 10 consecutive daily 60 min pavlovian training sessions, during which they were presented with two stimuli for 10 s, a tone (2.9 kHz, 5 dB), or two LED flashing lights. Each stimulus was present 16 times during the session. The order of stimulus presentations was randomly determined, and each stimulus trial was separated by a variable no stimulus intertrial interval (range 80–120 s; mean = 100 s). The stimuli were counterbalanced between mice; one associated with food delivery (CS+), and the other with no outcome (CS−). A single food pellet delivery occurred 5 s after CS+ onset (20 mg sweetened pellets; 5TUL, catalog #1811142, Test Diets).

Following training, two nose-poke detectors were added to the operant chamber on either side of the food magazine, each triggering presentation of either the CS+ or the CS− (right/left side of CS+ was counterbalanced among mice). Each CRf test session lasted 60 min, and the CS+ or CS− was delivered under a fixed ratio 1 (FR1) schedule of reinforcement. (One nose poke led to a single delivery of the conditioned reinforcer.)

In wild-type and α4^−/−^ mice, rates of nose-poke responses were first assessed in a baseline session, then again following cocaine (3, 10, 30 mg/kg, intraperitoneal (i.p.) injection) or saline in CRf test sessions on separate days and pseudorandomized in a Latin square design. Similarly CRf responding was assessed following saline or cocaine (10 mg/kg) administration in counterbalanced CRf sessions on separate days in adenovirus α4 knockdown and control virus-infused mice. Finally, cannulated wild-type, α4^−/−^ and conditional α4^D1−/−^ and α4^D2−/−^ mice underwent 4 test days in a Latin square design on separate days, during which they were administered intra-NAc infusions of either saline or THIP (3 mm) 20 min before an intraperitoneal injection of saline or cocaine (10 mg/kg), directly before testing.

When mice underwent multiple CRf test sessions, a 60 min refresher pavlovian conditioning session (as described above) was provided in the day between each CRf test session.

### Instrumental responding for primary reward

A separate group of mice from those used in CRf experiments was used to investigate instrumental responding for a primary reward. Following food deprivation to maintain ∼85% of baseline body weight, animals were trained in mouse operant chambers (Med Associates), each housed within a light-resistant, sound-attenuating cubicle. Operant chambers were set up in the same configuration as for CRf experiments, with two nose-poke detectors either side of a food magazine that could deliver 20 mg sweetened pellets (5TUL, catalog #1811142; Test Diets). In all sessions, nose pokes at one of the detectors (active) resulted in reinforcement (pellet delivery), whereas nose pokes at the other detector (inactive) resulted in no consequence. The side (right/left) of the active nose-poke detector was counterbalanced among mice. On the first day mice underwent a 15 h training session (including the dark phase), during which the reinforcer was available on an FR1 schedule. Subsequent sessions were daily 1 h sessions, during which the FR was 1, 2, or 4 for 3 consecutive sessions each. The total amount of nose-poke responses made at the active and inactive detectors was averaged across the three sessions for each FR schedule.

### Locomotor activity

A separate group of mice from those used in CRf and instrumental responding experiments was used to investigate locomotor activity. Mice were placed into circular runways. Locomotion was measured over 60 min in annular black Perspex runways (diameter 24 cm, cannula width 6.5 cm) placed atop a clouded Perspex sheet on an elevated frame. A digital camera positioned beneath the sheet captured the silhouettes of the edges of the boxes and the mice within them, which was then relayed to a computer to be recorded. A MATLAB (MathWorks) video analysis program and a Microsoft Excel macro converted the video data into a measure of the distance traveled in meters. Cannulated wild-type, α4^−/−^, and conditional α4^D1−/−^ and α4^D2−/−^ mice underwent 4 test days in a Latin square design, during which they were administered intra-NAc saline or THIP (3 mm) 20 min before an intraperitoneal injection of saline or cocaine (10 mg/kg), directly before testing.

### Quantitative reverse transcription PCR

RNA was extracted from biopsy punches (1 × 1 mm) of the NAc and dorsal striatum (RNeasy Mini Kit (QIAGEN) and the amount of RNA was determined using a NanoDrop 2000 microvolume spectrophotometer (Thermo Fisher Scientific). RNA (250 ng) was subjected to reverse transcriptase in the presence of oligo(dT), and 1 μl of the resulting cDNA (15 μl) was used in a qPCR reaction (SYBRGreen, Sigma-Aldrich) to amplify Gabra4 (CCACCCTAAGCATCAGTGC and CTGAATGGACCAAGGCATTT) and Gapdh (TGTCTCCTGCGACTTCAAC and AGCCGTATTCATTGTCATACC) as a reference gene. For each sample (run in triplicate) the cycle threshold was determined; dCT change from reference gene (gapdh), ddCt (change from Ad-NSS), and the fold change of expression from Ad-NSS-treated mice were calculated and analyzed according to the Pfaffl method (Pfaffl, 2001).

### Immunofluorescence

Mice were anesthetized with pentobarbitol sodium and phenytoin sodium solution (Euthasol), then transcardially perfused with 4% paraformaldehyde (Sigma-Aldrich). Fixed brains were removed, submerged in 30% sucrose in PBS for 72 h, then sliced on a microtome at 60 μm. Slices were incubated overnight in rabbit anti-GFP polyclonal primary antibody (1:10,000; Abcam), and then for 2 h in donkey anti-rabbit secondary antibody (1:300; Jackson ImmunoResearch). Images were acquired using a fluorescence microscope (Zeiss).

### Drugs

Cocaine hydrochloride was obtained from Macfarlan Smith. THIP (4,5,6,7-tetrahydroisoxazolo(5,4-c)pyridin-3-ol) was a gift from Bjarke Ebert (Lundbeck). Both drugs were dissolved in 0.9% saline, and administered intraperitoneally at an injection volume of 10 ml/kg and intracranially as described above.

### Experimental design and statistical analyses

qPCR data were analyzed using a three-way mixed factors ANOVA, with virus treatment and sex as between-subjects factors, brain region as a within-subjects factor, and fold change in Gabra4 mRNA expression from the Ad-NSS control virus group as the dependent variable. Pavlovian conditioning was assessed using four-way mixed factors ANOVAs, with genotype and sex as between-subjects factors, conditioned stimulus (CS+ or CS−) and session as within-subjects factors, and magazine entries made during presentation of the conditioned stimulus as the dependent variable. Reward collection latencies during pavlovian conditioning were assessed using three-way mixed factors ANOVAs, with genotype and sex as between-subjects factors, session as a within-subjects factor, and latency to collect the reward as the dependent variable. The dose–response of cocaine-potentiation of CRf data was analyzed using a four-way mixed-factors ANOVA, with genotype and sex as between-subjects factors, conditioned stimulus and drug dose as the within-subjects factors, and number of nose-poke responses per session as the dependent variable. Instrumental responding (primary reinforcement) was assessed using four-way mixed factors ANOVAs, with genotype and sex as between-subjects factors, nose-poke choice and fixed ratio schedule as within-subject factors, and nose-poke responses as the dependent variable. All other CRf data were analyzed using five-way mixed factors ANOVAs, with genotype and sex as the between-subjects factors, conditioned stimulus, intra-NAc infusion (THIP or saline), and intraperitoneal injection (cocaine or saline) as the within-subjects factors, and nose-poke responses as the dependent variable. *Post hoc* Bonferroni comparisons tests were performed when ANOVA main effects or interactions were significant (*p* < 0.05). Mauchly’s sphericity test was used to assess the assumption of sphericity, and the Greenhouse–Geisser correction was applied where necessary (Mauchly’s test *p* < 0.05). IBM SPSS Statistics software was used for all statistical analyses.

### Data availability

All data created during this research is openly available from the University of Sussex Research Data Repository Figshare at https://doi.org/10.25377/sussex.23987934.

## Results

### Increased conditioned reinforcement responding in GABA_A_R α4^−/−^ mice

To assess the role of α4-containing GABA_A_Rs in controlling instrumental responding for a conditioned reinforcer, we trained mice to associate one of two stimuli (either a tone or a flashing light, counterbalanced between animals) with a food pellet reward (CS+), whereas the other stimulus was paired with no outcome (CS−; Figs. 1*A*,*B*, top). Both wild-type (WT) and constitutive α4-subunit knock-out mice (α4^−/−^) were able to learn the reward-predictive properties of the CS+ to a similar extent, as indicated by increased and decreased approaches to the food delivery chamber on CS+ and CS− presentations, respectively (Fig. 1*C*; conditioned stimulus by session interaction; *F*_(9,144)_ = 31.34, *p <* 0.001). Similarly, although the latency to collect the reward decreased across pavlovian conditioning sessions, this decrease did not significantly differ between genotypes, indicating that the motivation to collect the reward was unaffected in both groups (Fig. 1*D*; significant main effect of session, *F*_(9,144)_ = 16.85, *p <* 0.001).

In a test of CRf responding, both genotypes showed significantly greater nose-poke responding for the CS+ than for the CS− (Fig. 1*B*, bottom, *E*; main effect of conditioned stimulus, *F*_(1,16)_ = 352.09, *p <* 0.001), demonstrating the reinforcing effect of the CS+. However, α4^−/−^ mice displayed a greater degree of instrumental responding for the CS+, but not the CS−, than WT controls (Fig. 1*E*; conditioned stimulus by genotype interaction; *F*_(1,16)_ = 31.65, *p <* 0.001). We then investigated whether cocaine could potentiate conditioned responding, as had previously been reported with cocaine and other psychostimulants (Robbins, 1975; Dixon et al., 2010). We found cocaine dose-dependently potentiated instrumental responding for the CS+, but not the CS−, to the same extent across genotypes, amplifying the initial cocaine-free pattern of responding (Fig. 1*F*; conditioned stimulus by genotype interaction, *F*_(1,16)_ = 42.44, *p <* 0.001; conditioned stimulus by cocaine dose interaction, *F*_(3,48)_ =37.13, *p <* 0.001). Thus, expression of α4-containing GABA_A_Rs appears to negatively modulate the ability for CRfs to drive instrumental responding.

### NAc knock down of α4-GABA_A_Rs augments conditioned reinforcement responding

**Figure 2.**
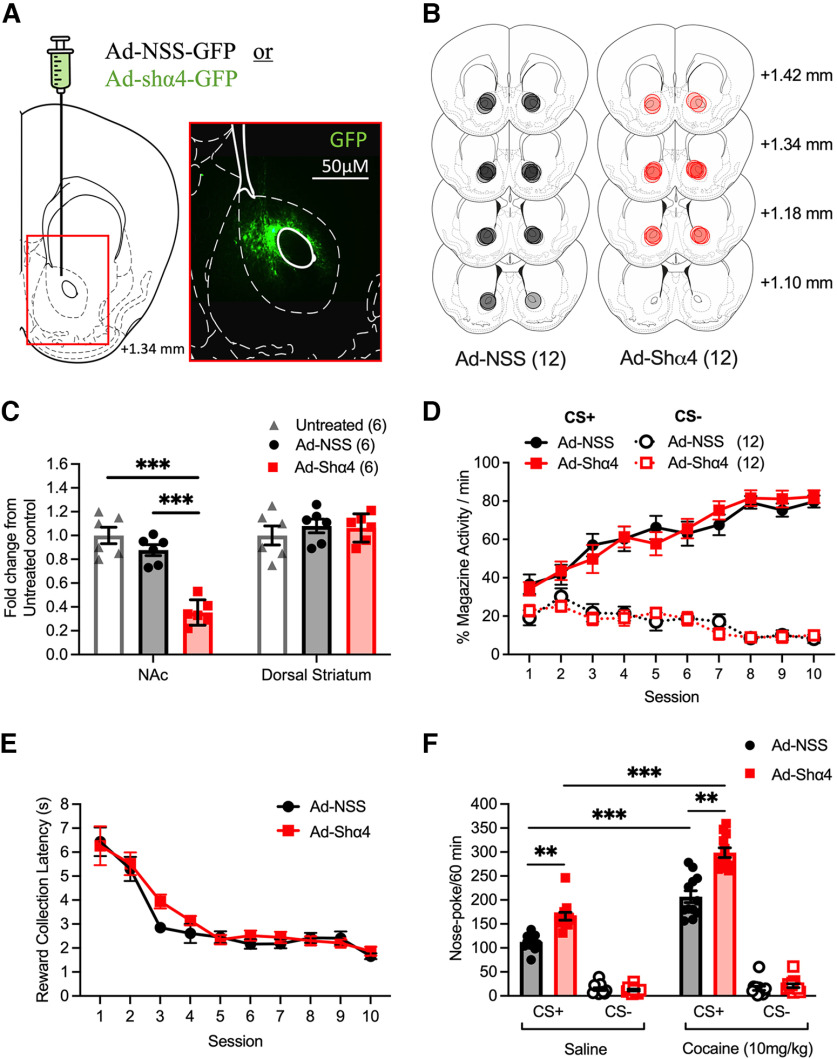
Effects of cocaine on instrumental responding for a conditioned reinforcer in control and α4-subunit viral knock-down mice. ***A***, Schematic of the infusion site for bilateral adenovirus delivery in the NAc (left). Immunohistochemical histologic analysis of virus expression in the region indicated by the red rectangle (right). Green areas in immunohistochemical image indicate anti-GFP antibody staining. Scale bar, 50 μm. ***B***, Schematic of Ad-NSS (scrambled control adenovirus, *n* = 12) and Ad-shα4 (knockdown of GABA_A_R α4-subunits, *n* = 12) expression sites in the NAc. Black and red circles indicate the maximum spread of the virus in each animal for Ad-NSS and Ad-shα4 viruses, respectively. ***C***, qRT-PCR quantification of mRNA from NAc and dorsal striatum tissue to confirm the specificity of Ad-shα4 adenovirus reduction of α4 mRNA expression in the NAc. Data show fold change expression of Gabra4 in mice infused with Ad-shα4 or Ad-NSS ± SEM compared with an untreated control group (*n* = 6 per group). ***D***, Pavlovian training; both Ad-NSS- and Ad-shα4-infused mice learned the association between a pavlovian cue and a food reward at the same rate. ***E***, The latency in seconds to collect the pellet reward during pavlovian training decreased across the 10 training sessions, with no difference observed between Ad-NSS- and Ad-shα4-infused mice. ***F***, Ad-shα4-infused mice showed significantly greater nose-poke responding for a reward-paired conditioned reinforcer (CS+) than Ad-NSS-infused mice. Responding was potentiated by cocaine (10 mg/kg, i.p.) administration to a similar extent in Ad-NSS- and Ad-shα4-infused mice. Data are presented as mean ± SEM; **p < 0.01, *** *p <* 0.001, *post hoc* Bonferroni multiple comparisons.

To investigate whether the augmentation of CRf responding observed in GABA_A_R α4^−/−^ mice was because of deletion of these subunits from the NAc, a region that has been implicated in the ability for conditioned reinforcers to modulate instrumental responding, we next knocked down GABA_A_R α4 specifically from the NAc. An adenovirus carrying a shRNA designed to knock down α4 (Ad-shα4; validated in Rewal et al., 2009) or a scrambled control adenovirus (Ad-NSS) was microinjected directly into the NAc of wild-type mice (Fig. 2*A*,*B*). In a separate group of mice from those used for behavioral experiments, it was first confirmed by RT-PCR that Ad-shα4, but not Ad-NSS or wild-type, mice demonstrated a reduction in GABA_A_R α4-subunit mRNA expression (66 ± 6.7% reduction) specifically within the NAc, but not the neighboring dorsal striatum (Fig. 2*C*; brain region by virus type interaction, *F*_(1,12)_ = 13.21, *p <* 0.001).

**Figure 3.**
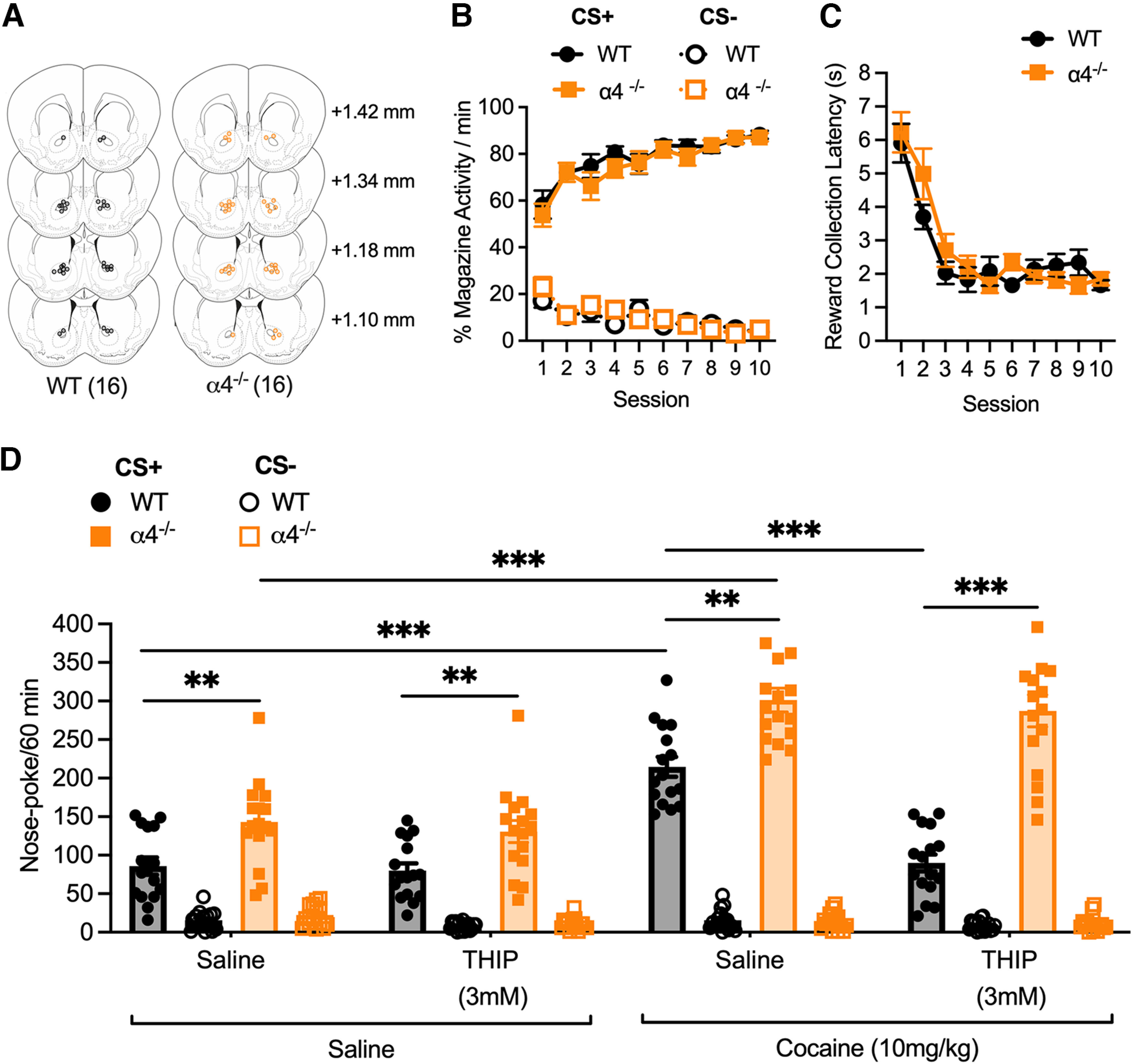
Effects of THIP on responding in conditioned reinforcement in WT and α4^−/−^ mice. ***A***, Cannula placements for intra-accumbal infusions of saline or THIP (3 mm) in WT (*n* = 16) and α4^−/−^ (*n* = 16) mice. Black and orange circles indicate the location of the end of the cannula in each animal in WT and α4^−/−^ mice, respectively. ***B***, Pavlovian training; WT and α4^−/−^ mice learned the association between the cue (CS+) and delivery of a food reward at the same rate. ***C***, The reward collection latency during pavlovian training decreased to a similar degree across the 10 training sessions in WT and α4^−/−^ mice. ***D***, Nose-poke responding for the CS+ following local NAc infusion of saline or THIP (3 mm) and injection of saline or cocaine (10 mg/kg, i.p.). WT but not α4^−/−^ mice displayed an attenuation of cocaine-potentiated CS+ responding following intra-NAc THIP infusion. Data are presented as mean ± SEM; **p < 0.01, ***p < 0.001 *post hoc* Bonferroni multiple comparisons.

**Figure 4.**
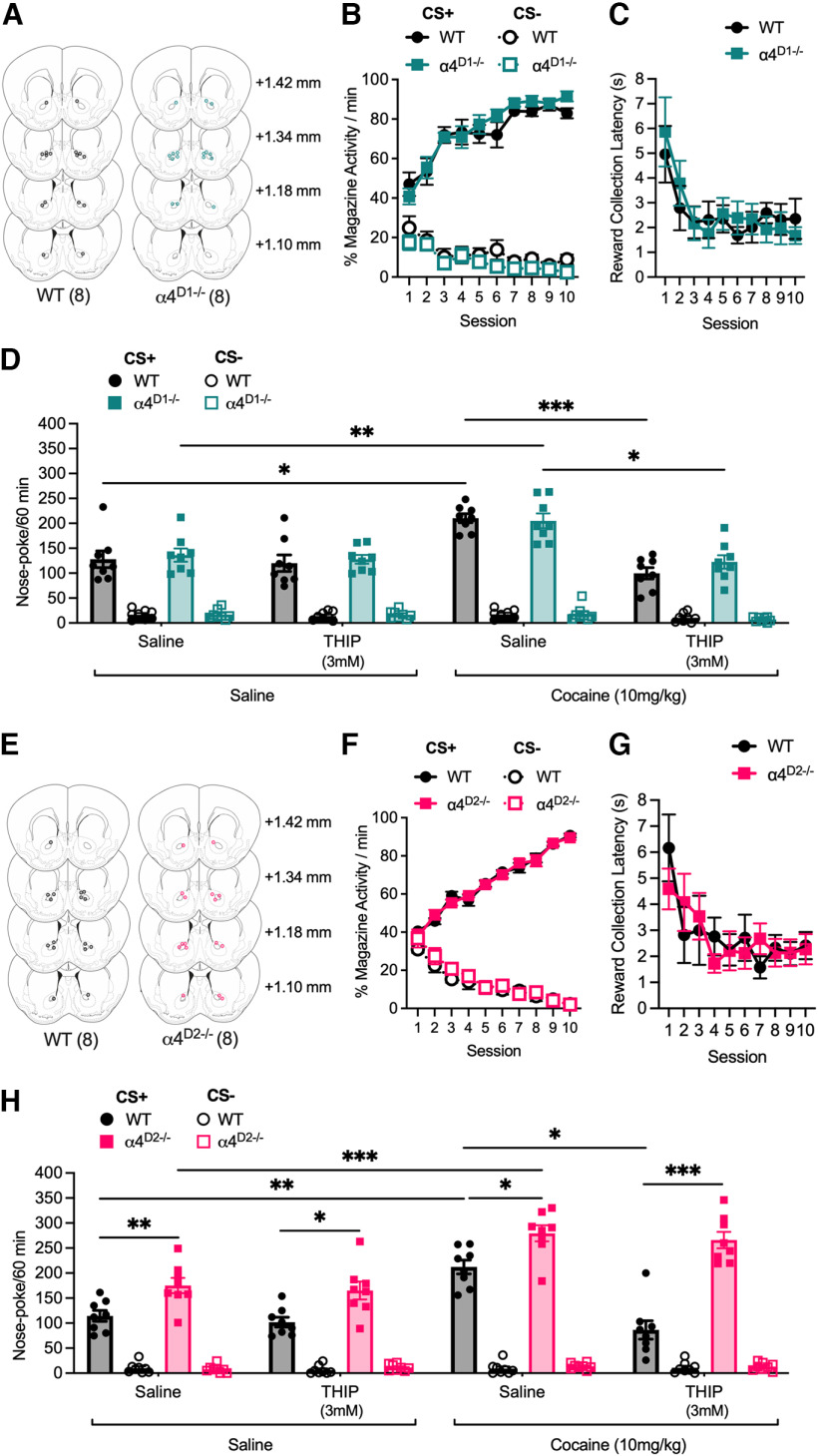
Effect of DRD1- or DRD2-specific ablation of α4-GABA_A_Rs on nose-poke responding for a conditioned reinforcer. ***A***, Cannula placements for intra-accumbal infusions of saline or THIP (3 mm) in WT (*n* = 8) and α4^D1−/−^ (*n* = 8) mice. Black and teal circles indicate the location of the end of the cannula in each animal in WT and α4^D1−/−^ mice, respectively. ***B***, Pavlovian training; WT and α4^D1−/−^ mice learned the association between the pavlovian cue and food reward to a similar extent. ***C***, The reward collection latency during pavlovian training decreased to a similar degree across the 10 training sessions in WT and α4^D1−/−^ mice. ***D***, No difference in CRf responding was observed between WT and α4^D1−/−^ mice and cocaine-potentiated responding to a similar magnitude in both genotypes. THIP alone had no effect on responding but blocked cocaine potentiation of CS+ responding in both genotypes. ***E***, Cannula placements for intra-accumbal infusions of saline or THIP (3 mm) in WT (*n* = 8) and α4^D2−/−^ (*n* = 8) mice. Black and pink circles indicate the location of the end of the cannula in each animal in WT and α4^D2−/−^ mice, respectively. ***F***, Pavlovian training; WT and α4^D2−/−^ mice learned the association between the pavlovian cue and food reward to a similar extent. ***G***, The reward collection latency during pavlovian training decreased to a similar degree across the 10 training sessions in WT and α4^D2−/−^ mice. ***H***, Instrumental responding for the CS+ was increased in α4^D2−/−^ mice compared with WTs. Cocaine potentiated responding for the CS+ to a similar degree in both genotypes. THIP alone had no effect on CS+ responding in either genotype; however, the attenuation of cocaine potentiation seen in WTs was absent in α4^D2−/−^ mice. Data are presented as mean ± SEM; *p < 0.05, ** *p <* 0.01, *** *p <* 0.001 *post hoc* Bonferroni multiple comparisons.

Both Ad-NSS and Ad-shα4 mice were able to learn the food-predictive properties of the CS+ during pavlovian conditioning, with both groups demonstrating similar increases and decreases in approaches to the food delivery chamber during CS+ and CS− trials, respectively (Fig. 2*D*; conditioned stimulus by session interaction; *F*_(9,180)_ = 28.83, *p <* 0.001). Additionally, the latency to collect the reward decreased to a similar degree in both virus groups (Fig. 2*E*; significant main effect of session, *F*_(9,180)_ = 33.71, *p <* 0.001). As with constitutive α4^−/−^ mice, in a CRf responding test, Ad-shα4 mice showed increased instrumental responding for the CS+, but not the CS−, relative to Ad-NSS controls (Fig. 2*F*; conditioned stimulus by virus interaction, *F*_(1,20)_ = 33.60, *p <* 0.001). Similarly, cocaine potentiated CRf responding for the CS+, but not the CS−, equally between virus groups (Fig. 2*F*; conditioned stimulus by drug group interaction, *F*_(1,20)_ = 133.93, *p <* 0.001). These data indicate that that α4-GABA_A_Rs within the NAc control the ability of conditioned reinforcers to elicit instrumental responses.

### Attenuated cocaine potentiation of conditioned reinforcement responding in THIP-treated α4 wild-type, but not α4^−/−^ mice

Given that constitutive deletion and NAc-specific knockdown of α4-GABA_A_Rs were able to augment instrumental responding for a conditioned reinforcer, we next investigated whether intra-NAc infusion of the GABA_A_R δ subunit-specific agonist THIP, which acts exclusively at α4βδ GABA_A_Rs within the NAc (Maguire et al., 2014), is able to reduce conditioned reinforcement responding.

In both α4^−/−^ and WT mice, indwelling cannulae were targeted at the NAc to enable local infusion of THIP or saline (Fig. 3*A*). As previously, α4^−/−^ and WT mice demonstrated a similar ability to learn the food-predictive properties of the CS+ during pavlovian conditioning (Fig. 3*B*; conditioned stimulus by session; *F*_(9,252)_ = 17.80, *p <* 0.001), and no differences between genotypes were observed for the latency to collect the reward during conditioning sessions (Fig. 3*C*; significant main effect of session, *F*_(9,252)_ = 29.01, *p <* 0.001).

Local infusion of THIP (3 mm, 210 ng per side) into the NAc did not alter baseline CRf responding for the CS+ or the CS− but was able to decrease cocaine-potentiated responding specifically for the CS+ in WT, but not α4^−/−^ mice (Fig. 3*D*; conditioned stimulus by infusion by injection by genotype interaction; *F*_(1,28)_ = 11.15, *p <* 0.001). The specificity of this attenuation to WT mice suggests that selective activation of NAc α4βδ GABA_A_Rs by THIP, which does not occur in α4^−/−^ mice (Maguire et al., 2014), modulates the ability of cocaine to potentiate instrumental responding for a conditioned reinforcer.

### Targeted deletion of GABA_A_ α4 subunits from DRD2 neurons increases responding for a conditioned reinforcer and blocks THIP attenuation of cocaine-potentiated responding

Given that our previous findings have suggested that α4-GABA_A_Rs on DRD1 or DRD2 neurons have distinct roles in mediating reward-associated behaviors (Maguire et al., 2014), we next explored the consequences of specifically deleting α4 subunits from DRD1 and DRD2 neurons (α4^D1−/−^ and α4^D2−/−^ mice, respectively) on CRf responding and its potentiation by cocaine. The α4^D1−/−^ and α4^D2−/−^ mice were created by crossing floxed α4 mice with DRD1=Cre and DRD2-Cre recombinase mice, respectively.

As with the previous experiments, indwelling cannulae were targeted at the NAc for local infusion of THIP or saline (Fig. 4*A*,*E*). No differences were seen between any of the genotypes in their ability to learn the reward-predictive properties of the CS+ during pavlovian conditioning (Fig. 4*B*; α4^D1−/−^ and WT, conditioned stimulus by session interaction, *F*_(9,108)_ = 16.26, *p <* 0.001; Fig. 4*F*; α4^D2−/−^ and WT, conditioned stimulus by session interaction, *F*_(9,108)_ = 120.43, *p <* 0.001). Similarly, no differences in the time to collect the reward during conditioning sessions were observed between genotypes (Fig. 4*C*; α4^D1−/−^ and WT, significant main effect of session, *F*_(1,108)_ = 4.19, *p <* 0.001; Fig. 4*G*; α4^D2−/−^ and WT, significant main effect of session, *F*_(9,108)_ = 3.95, *p <* 0.001).

In the CRf test, α4^D1−/−^ mice showed discriminative responding for the CS+ (over the CS−), which was at a similar level to that observed in WT mice (Fig. 4*D*; main effect of CS, *F*_(1,12)_ = 718.38, *p <* 0.001). As with constitutive α4^−/−^ and NAc viral knock-down Ad-Shα4 mice, cocaine was found to potentiate CRf responding for the CS+ to a similar degree in α4^D1−/−^ and WT mice (Fig. 4*D*; conditioned stimulus by intraperitoneal injection interaction, *F*_(1,12)_ = 8.19, *p =* 0.014). Additionally, intra-NAc infusion of THIP (3 mm) was able to attenuate cocaine potentiation of CRf responding for the CS+ in both α4^D1−/−^ and WT mice (Fig. 4*D*; CS by intraperitoneal injection by intra-NAc infusion interaction, *F*_(1,12)_ = 53.19, *p <* 0.001). Thus, α4-GABA_A_Rs on DRD1 neurons do not appear to contribute to CRf responding or its potentiation by cocaine.

In contrast, α4^D2−/−^ mice demonstrated increased CRf responding for the CS+, but not the CS−, compared with their WT counterparts (Fig. 4*H*; CS by genotype interaction, *F*_(1,12)_ = 91.33, *p <* 0.001). Additionally, although WT mice showed reduced cocaine potentiation of CRf responding specifically for the CS+ following intra-NAc infusion of THIP (3 mm), this effect was absent in α4^D2−/−^ mice (Fig. 4*H*; CS by intraperitoneal injection by intra-NAc infusion interaction by genotype interaction, *F*_(1,12)_ = 9.75, *p <* 0.01). 

These findings indicate that the augmentation of CRf responding, as well as the ability of THIP to block cocaine potentiation of CRf responding in constitutive α4^−/−^ mice is likely because of the absence of α4-GABA_A_Rs on DRD2-neurons. These data suggest an important role of α4-GABA_A_Rs on DRD2 neurons in controlling behavioral responses to conditioned reinforcers.

### Constitutive or DRD1-/DRD2-specific deletion of GABA_A_ α4 subunits has no effect on responding rates for a primary reinforcer

**Figure 5.**
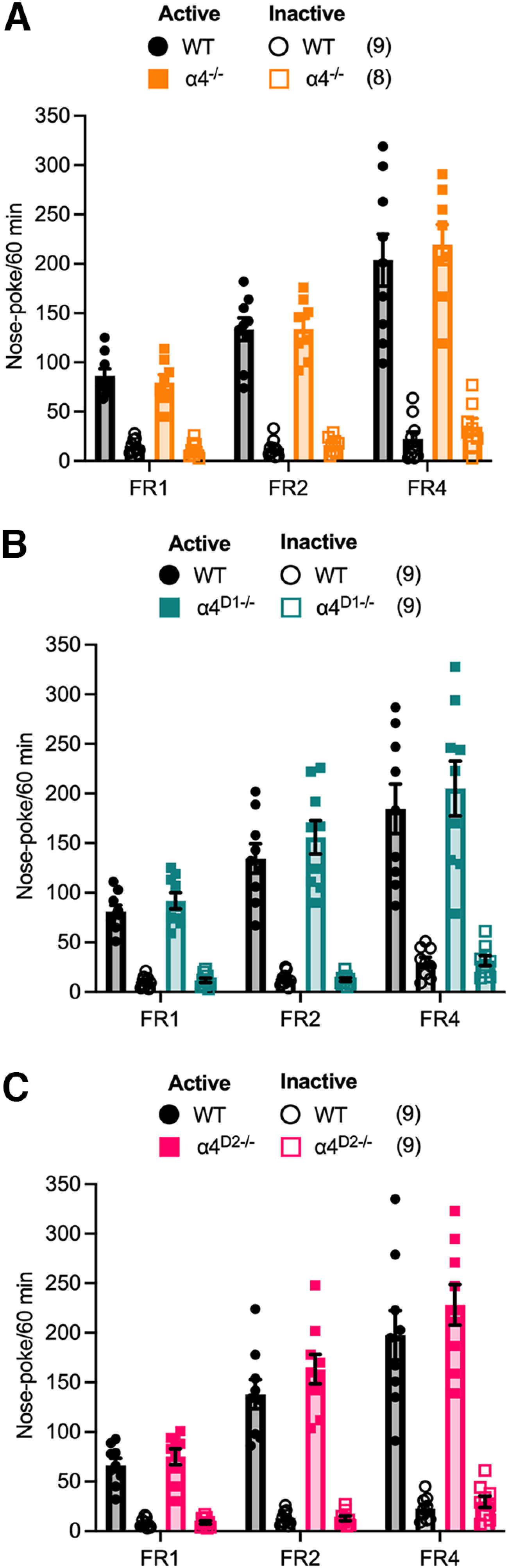
Effect of constitutive or DRD1- or DRD2-specific ablation of α4-GABA_A_Rs on instrumental responding for a primary reinforcer. ***A–C***, α4^−/−^ (***A***), α4^D1−/−^ (***B***), and α4^D2−/−^ (***C***) mice could be differentiated from their WT counterparts in their total amount of nose-poke responses for a primary reinforcer under FR1, FR2, or FR4 schedules of reinforcement in 60 min sessions. Data are presented as mean ± SEM.

It is possible that increased cue responding is actually the result of a more general increase in instrumental responding. Thus, to eliminate this possibility, we tested whether instrumental responding for a primary reinforcer (10% sucrose) on ascending FR schedules was also altered in constitutive and conditional knockouts. Although mice demonstrated discriminative responding at the active nose-poke detector that increased across ascending FR schedules, the number of nose pokes for the active nose poke did not differ between genotypes across any of the FR schedules (Fig. 5*A*; α4^−/−^ and WT, nose poke by FR interaction, *F*_(2,26)_ = 23.68, *p <* 0.001; Fig. 5*B*; α4^D1−/−^ and WT, nose poke by FR interaction, *F*_(2,28)_ = 21.09, *p <* 0.001; Fig. 5*C*; α4^D2−/−^ and WT, nose poke by FR interaction, *F*_(2,28)_ = 21.22, *p <* 0.001). Thus, the increase in instrumental responding for the CRf in α4^−/−^ and α4^D2−/−^ mice cannot be explained by altered instrumental learning.

### Constitutive or DRD1-/DRD2-specific deletion of GABA_A_ α4 subunits has no effect on locomotor activity

**Figure 6.**
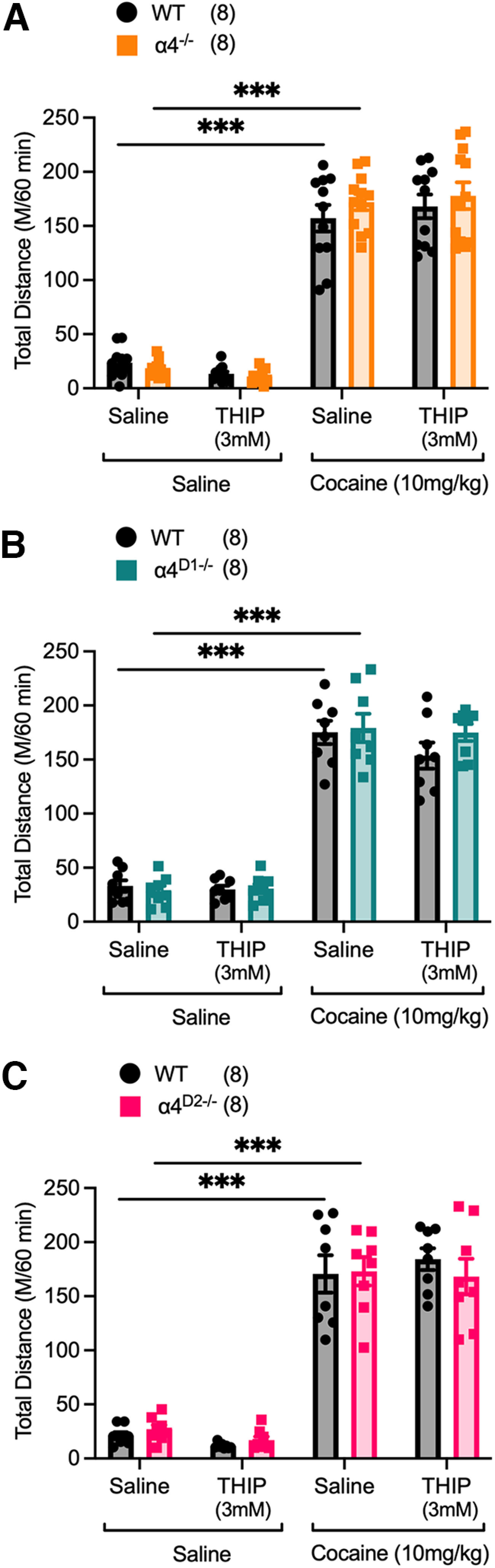
***A–C***, Effect of constitutive or DRD1- or DRD2-specific ablation of α4-GABA_A_Rs on locomotor activity. α4^−/−^ (***A***), α4^D1−/−^ (***B***), and α4^D2−/−^ (***C***) mice did not significantly differ from their WT counterparts in the total distance traveled in locomotor runways when administered saline or cocaine (10 mg/kg, i.p.) in 60 min sessions. Neither intra-NAc infusion of saline or THIP (3 mm) had any effect on baseline (intraperitoneal saline) or cocaine (10 mg/kg, i.p.)-potentiated locomotion in all genotypes. Data are presented as mean ± SEM; *** *p <* 0.001 *post hoc* Bonferroni multiple comparisons.

Finally, to eliminate the possibility that altered responding for CRf is because of changes in locomotor activity, we measured baseline (saline intraperitoneal injection) and 10 mg/kg, i.p., cocaine-induced locomotor activity in α4^−/−^, α4^D1−/−^, and α4^D2−/−^ mice given intra-NAc infusions of saline or THIP (3 mm). In all mice cocaine was found to increase the total distance traveled over a 60 min session (Fig. 6*A*; α4^−/−^ and WT, significant main effect of intraperitoneal injection, *F*_1,19)_ = 567.58, *p <* 0.001; Fig. 6*B*; α4^D1−/−^ and WT, significant main effect of intraperitoneal injection, *F*_(1,12)_ = 905.59, *p <* 0.001; Fig. 6*C*; α4^D2−/−^ and WT, significant main effect of intraperitoneal injection, *F*_(1,12)_ = 488.90, *p <* 0.001), However, this potentiation of locomotion did not significantly differ between genotypes. Additionally, it was found that intra-NAc infusion of THIP did not alter locomotion in α4^−/−^, α4^D1−/−^, α4^D2−/−^, or their WT counterparts when compared with sessions in which saline was infused. Thus, augmentation of instrumental responding for the CRf in α4^−/−^ and α4^D2−/−^ mice and the intra-NAc THIP-induced decrease in cocaine potentiation of CRf responding are not the result of altered locomotion but rather reflect changes in the ability of CRfs to drive behavioral responses.

## Discussion

**Figure 7.**
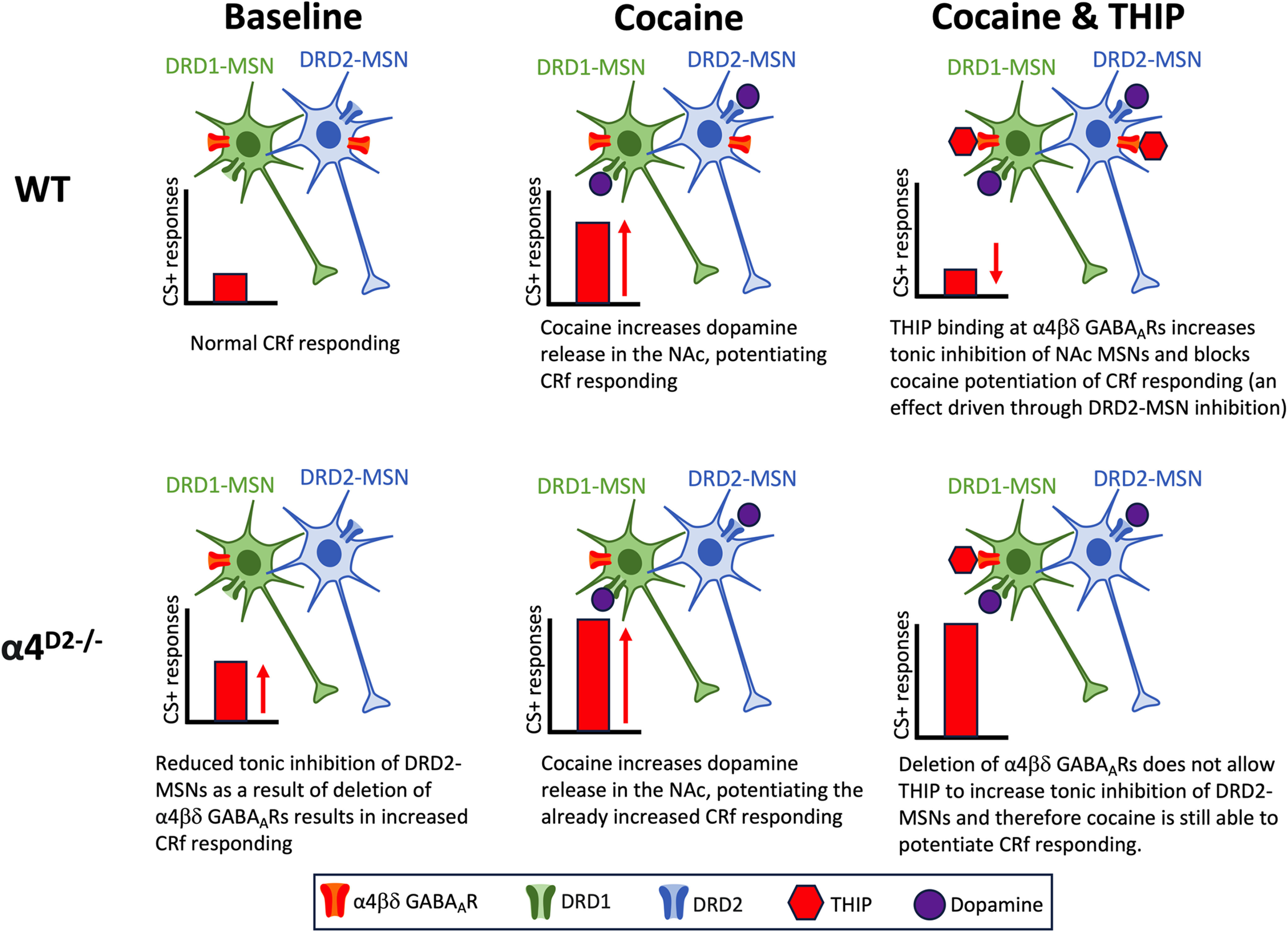
Schematic of theoretical nucleus accumbens cellular mechanisms controlling instrumental responding for a conditioned reinforcement (CRf), its potentiation by cocaine, and the actions of THIP on cocaine-potentiated CRf responding in WT and α4^D2−/−^ mice.

The data presented here demonstrate that a global deletion of α4-GABA_A_Rs increases instrumental responding for a conditioned reinforcer but, importantly, not for a conventional reward. Furthermore, although activation of α4βδ-GABA_A_Rs by THIP was not able to reduce baseline CRf responding, it did block cocaine-potentiated responding when infused directly into the NAc, an effect that was abolished in α4^−/−^ mice. The localization of these α4-GABA_A_R actions to the NAc was subsequently confirmed by the observation of an increased CRf response comparable to that in α4^−/−^ mice following viral knockdown of gabra4 expression within the NAc. Finally, the interaction of α4-GABA_A_R-mediated inhibition with distinct striatal cell types was explored with the use of DRD1- and DRD2-neuron-specific GABA_A_R α4-subunit knock-out mice. The increased CRf responding in constitutive α4-GABA_A_R knock-out and viral knock-down mice was replicated only in DRD2-neuron-specific α4-GABA_A_R knock-out mice, and the ability of intra-accumbal THIP to reduce cocaine-potentiated CRf responding was similarly lost in these mice (Fig. 7).

Constitutive knock-out, cell-specific knock-out, and NAc-specific viral knock-down mice were able to learn the reward-predictive properties of a conditioned cue (discriminated approach to food magazine) to the same extent as their respective controls in all experiments, indicating that α4βδ-GABA_A_Rs play little role in learning the predictive qualities of food-conditioned cues. All mice also showed similar levels of instrumental responding for a primary reinforcer, consistent with previous evidence of unchanged responding for sucrose following α4 or δ (paired with α4 in α4βδ GABA_A_Rs in the NAc) knockdown in the NAc of rats (Rewal et al., 2009; Nie et al., 2011). Thus, α4-GABA_A_Rs appear not to be involved in pavlovian associative learning processes or instrumental responding but, rather, modulate the expression of behavioral responses specifically to conditioned stimuli.

Removal of tonic GABA inhibition of DRD2 neurons (by deletion of α4-GABA_A_R) causes an increase in responding for a conditioned reinforcer, suggesting that activation of DRD2 neurons potentiates CRf responding (Fig. 7*A*). This interpretation is consistent with previous evidence that low doses of dopamine DRD2 antagonists sulpiride and raclopride both facilitate CRf responding (Smith et al., 1997), although it is important to note that the same study reported higher doses of the same D2 antagonists to result in a reduction in CRf responding. The current experiments also indicate that the NAc is the site of action of DRD2-neuron-mediated potentiation of CRf responding. Mice with a viral knockdown of α4-subunits localized to the NAc demonstrate a similar phenotype to constitutive and DRD2-neuron-specific α4-GABA_A_R knock-out mice. Finally, the potentiation of CRf responding by psychostimulants also appears to be mediated by DRD2 neurons within the NAc. In the current experiments, intra-accumbal THIP was able to block the cocaine potentiation of CRf responding in wild-type mice but not in either their counterpart constitutive nor DRD2-neuron-specific α4-GABA_A_R knock-out mice.

An interesting finding of our experiments was that THIP was unable to alter CRf responding in the absence of cocaine potentiation. This result may be explained by complex interactions between DRD2 agonism and α4-GABA_A_R-mediated tonic GABA currents within the NAc. Our previous work revealed that dopamine agonism is to reduce tonic GABAergic currents in NAc DRD2 neurons but only when DRD2 was agonized for an extended period, as might be expected following cocaine administration (Maguire et al., 2014). It is possible that under baseline conditions (in the absence of cocaine), tonic GABA inhibitory currents are maximal and would be unchanged by THIP administration. However, when cocaine is administered, tonic GABA currents are reduced, and THIP is thus able to increase this tonic GABA current and block the potentiation of CRf responding. In future, in vivo electrophysiological experiments may help to test this hypothesis.

Different subregions of the NAc have been reported to control different aspects of CRf responding. Indeed, although lesion of the NAc core or shell regions did not affect discriminated CRf responding (preferential instrumental responding for the CS+ over the CS−) under baseline conditions, NAc core lesion disrupted discriminated CRf responding when responding was potentiated by amphetamine, whereas NAc shell lesion blocked amphetamine potentiation of CRf responding (Parkinson et al., 1999). In the current study, although viral knockdown of GABA_A_R α4 subunits and intracranial infusions of THIP into the NAc primarily targeted the NAc core, it is possible that these manipulations also reached and affected the NAc shell. If this is the case, then THIP attenuation of cocaine potentiation of CRf responding could be explained by α4-GABA_A_R-mediated inhibition of NAc shell DRD2 neurons. Our evidence that intra-NAc THIP infusions, which inhibit NAc DRD1 and DRD2 neurons, do not alter CRf responding under baseline conditions also appears to match with the findings of Parkinson et al.’s (1999) study that lesions of the core or shell do not alter baseline CRf responding. Finally, although it was also previously reported that lesions of the NAc core impair discriminated pavlovian approach during pavlovian conditioning sessions, here, we found no evidence that knockdown of GABA_A_R α4 subunits within the NAc could improve pavlovian conditioning, unlike CRf responding, which was augmented by relief of NAc neurons from tonic GABAergic inhibition. It remains to be investigated whether intra-NAc infusions of THIP are able to impair pavlovian conditioning.

The findings of the current study also indicate that there are dissociable roles for α4-GABA_A_Rs on DRD1-NAc or DRD2-NAc MSNs in mediating various aspects of reward. Previously we have demonstrated that α4-GABA_A_Rs on DRD1 but not DRD2 neurons are involved in the expression of cocaine-CPP and its potentiation by cocaine (Maguire et al., 2014). Thus, we have uncovered a possible dichotomy between the striatal pathways involved in controlling behavioral responses to discrete (CRf) and contextual (CPP) conditioned cues. These findings add to a growing literature indicating that DRD2 neurons in the NAc also participate in the control of reward learning and motivation alongside their DRD1 neuron counterparts (Nishioka et al., 2023; Soares-Cunha et al., 2016a,b, 2019).

Finally, Although our data indicate that a relief of tonic inhibition of NAc DRD2-MSNs may underlie the augmentation of CRf responding, as well as cocaine-induced potentiation of CRf responding, it should be noted that our manipulation was not specific to MSNs. Indeed, it is known that DRD2 is also present on cholinergic interneurons within the striatum (Alcantara et al., 2003). In the future, manipulations that are specific to either DRD2-MSNs or cholinergic interneurons, such as those enabled by the use of adenosine a2a receptor and ChAT cre recombinase mouse lines, may help to further elucidate which specific neuron types contribute to CRf responding and its potentiation by cocaine. Additionally, further elucidation of the precise neural circuitry controlling CRf responding is necessary. Although our data indicate that NAc DRD2 neurons mediate CRf responding, it is still unclear whether this is achieved by GABAergic modulation of DRD2-MSNs projecting to the ventral pallidum, modulated lateral inhibition of DRD1-MSNs by DRD1-MSNs, or modulated DRD2-expressing cholinergic interneuron inhibition of MSNs in the NAc.

In conclusion, our results indicate that α4-GABA_A_R inhibition of DRD2 neurons, likely within the NAc, is a critical mechanism for controlling the expression of the reinforcing properties of discrete reward-conditioned cues.

## Synthesis

Reviewing Editor: Christine Portfors, Washington State University Decisions are customarily a result of the Reviewing Editor and the peer reviewers coming together and discussing their recommendations until a consensus is reached. When revisions are invited, a fact-based synthesis statement explaining their decision and outlining what is needed to prepare a revision will be listed below. The following reviewer(s) agreed to reveal their identity: NONE. Note: If this manuscript was transferred from JNeurosci and a decision was made to accept the manuscript without peer review, a brief statement to this effect will instead be what is listed below.

Thank you for responding so thoroughly to the reviewers’ comments.
